# Pharmacometabolomics uncovers key metabolic changes in the first-in-human study of β-lapachone derivative

**DOI:** 10.1007/s11306-025-02332-1

**Published:** 2025-08-19

**Authors:** Yeonseo Jang, Jihyun Kang, Yufei Li, Woori Chae, Eunsol Yang, SeungHwan Lee, Joo-Youn Cho

**Affiliations:** 1 Department of Clinical Pharmacology and Therapeutics, Seoul National University College of Medicine and Hospital, 101 Daehakro, Jongno-gu, Seoul, 03080 Korea; 2 Department of Biomedical Sciences, Seoul National University College of Medicine and Hospital, Seoul, 03080 Korea; 3 Kidney Research Institute, Seoul National University Medical Research Center, Seoul, 03080 Korea; 4 Seoul National University, Seoul, 08826 Republic of Korea; 5 Department of Chemistry, Washington University in St.Louis, St.Louis, MO USA; 6 Department of Bioengineering and Therapeutic Sciences, University of California, San Fransisco, San Fransisco, CA USA

**Keywords:** WK0202, NQO1, Nrf2, Metabolites, Arginine biosynthesis

## Abstract

**Introduction:**

WK0202, a β-lapachone derivative under clinical development, activates NAD(P)H quinone dehydrogenase 1 (NQO1), acting as a detoxifying and antioxidant agent. In this study, a metabolomics investigation of β-lapachone derivatives in humans is performed to characterize drug-induced alterations in endogenous metabolic pathways.

**Objectives:**

This study investigated metabolic alterations induced by WK0202 administration and their potential association with its therapeutic mechanism and efficacy. Using targeted and untargeted metabolomics approaches, we identified potential pharmacodynamic biomarker candidates that may reflect the drug’s activity and metabolic effects.

**Methods:**

Plasma samples from healthy subjects who received multiple doses of WK0202 were compared with a placebo control group. The metabolomic profiles were compared pre- and post-dose to identify significant metabolic changes. Significant metabolites were identified using statistical analyses, focusing on key metabolic pathways. To further investigate NQO1 genotype effects, Spearman correlation analysis was performed between post/pre-dose concentration ratios and genotypes.

**Results:**

Following WK0202 administration, significant changes were observed in the alanine, aspartate and glutamate metabolism, arginine biosynthesis, and lipid metabolism. Although most metabolites were not strongly dependent on NQO1 genotype or dose group, they exhibited an overall consistent trend. These alterations were indicative of Nrf2 pathway activation, possibly by NQO1-mediated drug activity.

**Conclusion:**

These metabolic alterations highlight the potential of endogenous metabolites as surrogate markers for identifying novel therapeutic targets and assessing the efficacy of WK0202 in future clinical studies.

**Supplementary Information:**

The online version contains supplementary material available at 10.1007/s11306-025-02332-1.

## Introduction

β-lapachone, derived from the bark of the lapacho tree, has long intrigued researchers owing to its unique capabilities (Yang et al., 2017). β-lapachone activates NQO1, a key enzyme regulated by the Nrf2 pathway that protects cells from oxidative stress and inflammation (Ngo & Duennwald, 2022; Qiu et al., 2022). However, NQO1 activity varies due to genetic polymorphisms. Particularly, the NQO1*2 variant destabilizes the enzyme and reduces its function (Nebert et al., 2002). These genetic differences alter β-lapachone metabolism, leading to variability in therapeutic efficacy and potential toxicity. Despite its therapeutic promise, β-lapachone’s toxicity, including DNA damage (Lima et al., 2024), limits its clinical application.

To overcome these limitations, β-lapachone derivatives, which mimic the structure of β-lapachone, have been actively developed with the goal of reducing toxicity while enhancing efficacy and safety. WK0202 is one of the β-lapachone derivatives that activates NQO1, leading to an increased nicotinamide adenine dinucleotide (NAD+) levels in the cytoplasm, as well as activation of Sirtuin1. Increased NAD + levels have anti-inflammatory and antioxidant functions (Kang et al., 2021; Rajman et al., 2018). Sirtuin1 also exerts anti-inflammatory responses by promoting p65 deacetylation and inhibiting Nuclear factor kappa B (NF-κB) activity (Yang et al., 2012). These mechanisms demonstrate the potential of WK0202 to alleviate cancer-related side effects, such as inflammation caused by anticancer agents (Oh et al., 2014).

Preclinical studies have demonstrated that WK0202 alleviates cancer-related side effects, including neutropenia and fatigue syndrome, by activating NQO1. In murine models, WK0202 effectively preserves neutrophil levels and mitigates inflammation-associated fatigue by regulating the NAD+/NADH balance, Sirtuin1 activation, and AMPK signaling. Although the mechanism of NQO1 activation mediated by β-lapachone is well-studied, the systemic effects of β-lapachone and its derivatives remain limited, particularly regarding their impact on endogenous metabolic networks. Previous studies have primarily employed cell-based systems, restricting their physiological relevance. In contrast, analyzing human clinical samples offers a more clinically relevant perspective on metabolic changes caused by β-lapachone derivatives. Additionally, pharmacometabolomics facilitates the development of new therapeutic strategies, with the potential to provide a deeper understanding of WK0202’s mechanism of action (Wu et al., 2021).

In the current study, a pharmacometabolomics approach was used to characterize metabolic changes following WK0202 administration to humans. Plasma samples were analyzed with targeted and untargeted metabolomics to elucidate the mechanism of WK0202 in a first-in-human context and propose potential pharmacodynamic biomarker candidates.

## Materials and methods

### Study design and subjects

Overall, 27 healthy volunteers participated in this study. The inclusion criteria for healthy volunteers were as follows: healthy adults aged 19–45 years at the time of screening, with a body weight of 50 kg or more and a body mass index (BMI) between 18.0 and 27.0. Participants with abnormal laboratory test results or medical conditions that could interfere with the study outcomes were excluded. The exclusion criteria included elevated aspartate transaminase (AST), alanine aminotransferase (ALT), total bilirubin, or creatine phosphokinase (CPK) levels (> 1.5 times the upper normal limit), an estimated glomerular filtration rate (eGFR) of < 90 mL/min/1.73 m², or positive serological tests for hepatitis B, hepatitis C, or human immunodeficiency virus (HIV).

Participants received three different doses (100 mg (*n* = 8), 200 mg (*n* = 6), 400 mg (*n* = 7)) of WK0202 and placebo (*n* = 6) once daily for 14 days. WK0202 or placebo was administered orally with 200 mL of water in a fasted state in the morning. No concomitant medications were administered during the study period except when deemed necessary for managing adverse reactions. Additionally, all participants consumed the same standardized meal. Blood samples (4 mL each) were collected pre-dose and post-dose (15 d 0 h) from subjects who had completed the 14-day medication regimen. The samples were analyzed using targeted and untargeted metabolomics approaches. The collected blood was stored in EDTA K2 tubes and centrifuged at 1977 rcf at 4 ℃ for 10 min. Plasma samples were then stored at −80 °C until use.

Plasma from healthy volunteers was provided by Seoul National University Hospital. Written informed consent was obtained from all participants for the use of human-derived materials in metabolite analysis (CRIS Registration Number: KCT0004830, Institutional Review Board No.: H-1911-103-1081).

### Chemicals and reagents

WK0202’s chemical structure is dunnione (2,3-dihydro-2,3,3-trimethylnaphtho[1,2-b] furan-4,5-dione) (Fig.**S1**) and it was synthesized by the Daegu-Gyeongbuk Medical Innovation Foundation (DGMIF). Water, acetonitrile (ACN, 99.9%), and methanol (MeOH, 99.8%) of HPLC grade were purchased from J.T. Baker (Phillipsburg, NJ, USA), and formic acid (95%) was purchased from Sigma-Aldrich (St. Louis, MO, USA).

### Targeted metabolomics

Samples were extracted using a 96-well plate Absolute IDQ p180 kit (BIOCRATES Life Science AG, Innsbruck, Austria) and prepared according to the manufacturer’s instructions, followed by analyzing with an ACQUITY UPLC system (Waters, Milford, MA, USA) coupled to a Triple Quad 5500 mass spectrometer (AB Sciex, Redwood City, CA, USA).

Briefly, plasma samples were dispensed onto the filter in the upper 96-well plate kit at a volume of 10 µL per well, followed by drying using a nitrogen evaporator. Subsequently, 50 µL of a 5% phenylisothiocyanate solution was added to derivatize the amino acids and biogenic amines. After incubation, the filter spots were again dried using a nitrogen evaporator. Metabolites were then extracted with 300 µL of 5 mM ammonium acetate in methanol and transferred to the lower 96-deep well plate via centrifugation. These extracts were further diluted with 600 µL of the MS running solvent for subsequent MS analysis.

Internal standards were used to ensure accurate metabolite quantification of metabolites and enhanced reliability of the detection process. Metabolites were detected using the multiple reaction monitoring mode. Data were normalized using pooled quality control (QC) samples, which were prepared by combining 50 µL aliquots from each individual sample, through MetIDQ^®^ (BIOCRATES Life Science AG, Innsbruck, Austria). For each individual metabolite, missing values were replaced with the limit of detection (LOD) according to the specific applicability of each metabolite. Data filtering was performed by excluding analytes for which more than 75% of the values were below the LOD. Consequently, statistical analysis was conducted using only 153 of the 188 metabolites, and all other LOD values were replaced with zero.

### Untargeted metabolomics

Sample preparation was achieved by protein precipitation with 400 µL of ice-cold extraction solution (methanol: acetonitrile = 1:1) added to 100 µL of each plasma sample. The mixture was vortexed for 5 min at room temperature and centrifuged at 18,341 rcf at 4 ℃ for 10 min. Supernatants were transferred to high-performance liquid chromatography vials for untargeted metabolomic analyses. Four microliters of each sample was injected into an ultra-high-performance liquid chromatography (UPLC) system equipped with a Q Exactive™ Plus Hybrid Quadrupole-Orbitrap™ Mass Spectrometer (Thermo Fisher Scientific, Bremen, Germany). Analytical quality was improved based on previously described quality assurance and control measures (Lippa et al., 2022; Mosley et al., 2024). Specifically, the injection order was randomized, and data correction and system suitability were verified using pooled QC samples.

An ACQUITY UPLC HSS T3, 1.8 μm x 100 mm column (Waters Corporation, Milford, MA, USA) was connected to the UPLC system for metabolite separation. For the mobile phase, water containing 0.1% formic acid (mobile phase A) and MeOH with 0.1% formic acid (mobile phase B) were used under gradient conditions. Gradient elution started with 5% B at 1 min, increased to 95% B at 6 min, and 98% B at 12 min. This was maintained for up to 22 min and then reduced to 5% B at 25 min. The flow rate was 0.4 mL/min, and the column temperature was maintained at 50 ℃. Analytes were detected with the following settings: Sheath Gas, 3 Arb; Aux Gas, 2 Arb; Sweep Gas, 0 Arb; Ion Transfer Tube Temperature, 320 °C; Vaporizer Temperature, 0 °C; Resolution, 120,000; Scan range, 100–1000 m/z.

**Fig. 1:**
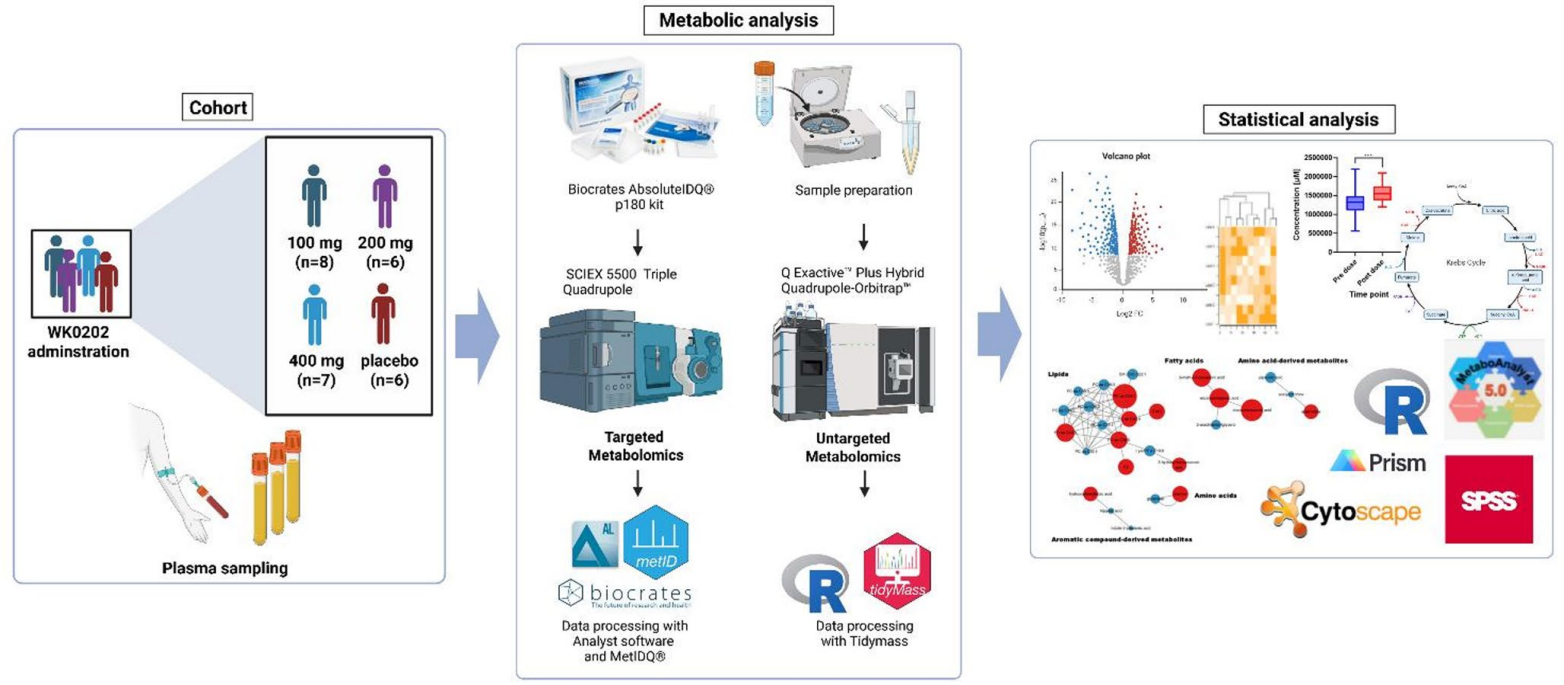
Workflow of targeted and untargeted metabolomics

All samples were analyzed under the mass scan mode (MS1), whereas QC samples were analyzed under both MS1 and data-dependent mass acquisition (ddMS2) modes. The MS1 and MS2 data were collected and converted to.mzXML and.mgf formats, respectively, using ProteoWizard 3.0.21256. Tidymass was used for raw data processing and metabolite annotation (Shen et al., 2022). Retention time correction and feature filtration were performed using the pooled QC samples, and baseline QC was utilized to exclude drug-related signals and their metabolites from analyses. Metabolite annotation was performed by cross-referencing with several open-source databases based on the MS1 and MS2 data. MS2 matching plots were used to verify the compound spectra, errors, and match scores for metabolite identification. We analyzed several available authentic compounds to compare the spectra for identification at Metabolomics standard initiative (MSI) level 1 and matched the MS2 spectra and library for identification at MSI level 2 (Reisdorph et al., 2019). The similarity of the identified compounds was confirmed using both MSI level 1 and level 2 methodologies. The workflow of targeted and untargeted metabolomics is shown in Fig. 1.

### Statistical analysis

The demographics among the treatment groups were compared using one-way ANOVA in IBM SPSS Statistics 26; a p-value < 0.05 was deemed statistically significant. Permutational multivariate analysis of variance (PERMANOVA) was performed using the adonis2 function in the vegan package (R version 4.4.1) to evaluate differences in the metabolomic profiles among groups. The analysis was conducted on a distance matrix computed using the Euclidean method, with 999 permutations to assess statistical significance. Statistical analysis of the metabolomic data was performed using MetaboAnalyst 5.0, and data were normalized through log transformation and Pareto scaling. Significant metabolites were determined by pairwise comparisons using a paired t-test with a false discovery rate (FDR)-adjusted p-value less than 0.05. To focus on drug-specific effects, we identified metabolites that showed significant changes between pre-dose and post-dose in the WK0202-treated groups (paired t-test, FDR-adjusted *p* < 0.05). Metabolites that also changed in the placebo group were excluded from further analysis. These metabolites were identified based on their retention times, concentrations, and relative intensities. Principal component analysis (PCA) score plots of metabolite distributions classified as pre-dose and post-dose were generated. The concentrations and relative intensities of significant metabolites between pre-dose and post-dose were standardized by Pareto scaling of features and samples, followed by hierarchical metabolite clustering using the Euclidean distance and Ward method. Network analysis was conducted by mapping onto MetaMapp (Barupal et al., 2012) and visualization with Cytoscape 3.9.2. Enriched metabolite sets between pre-dose and post-dose in both targeted and untargeted analyses were identified using the KEGG-based database provided by MetaboAnalyst 5.0. Subsequently, spaghetti plots, box plots, and volcano plots were visualized using GraphPad Prism 10.0 software.

## Results

### Subject demographics

Table**S1** shows the demographic characteristics of the study population. The participants were categorized into four groups: WK0202 administered at doses of 100 mg, 200 mg, and 400 mg, and a placebo group. Among the NQO1 genotypes, the *2/*2 group had the fewest participants. All subjects were male, and there were no statistically significant differences in age, height, or BMI among the groups.

### Targeted metabolomic profiling

Of the 188 metabolites included in the Biocrates p180 kit, 153 were successfully detected in plasma and used for PCA. PCA plots revealed the distribution of metabolic profiles at pre- and post-dose time points in subjects who received WK0202 at different doses (100 mg, 200 mg, or 400 mg) (Fig. S2a). The Kruskal–Wallis test identified no significantly altered metabolites, and the PERMANOVA analysis yielded a p-value of 0.527, indicating no significant differences in metabolic profiles among the dose groups.

**Fig. 2:**
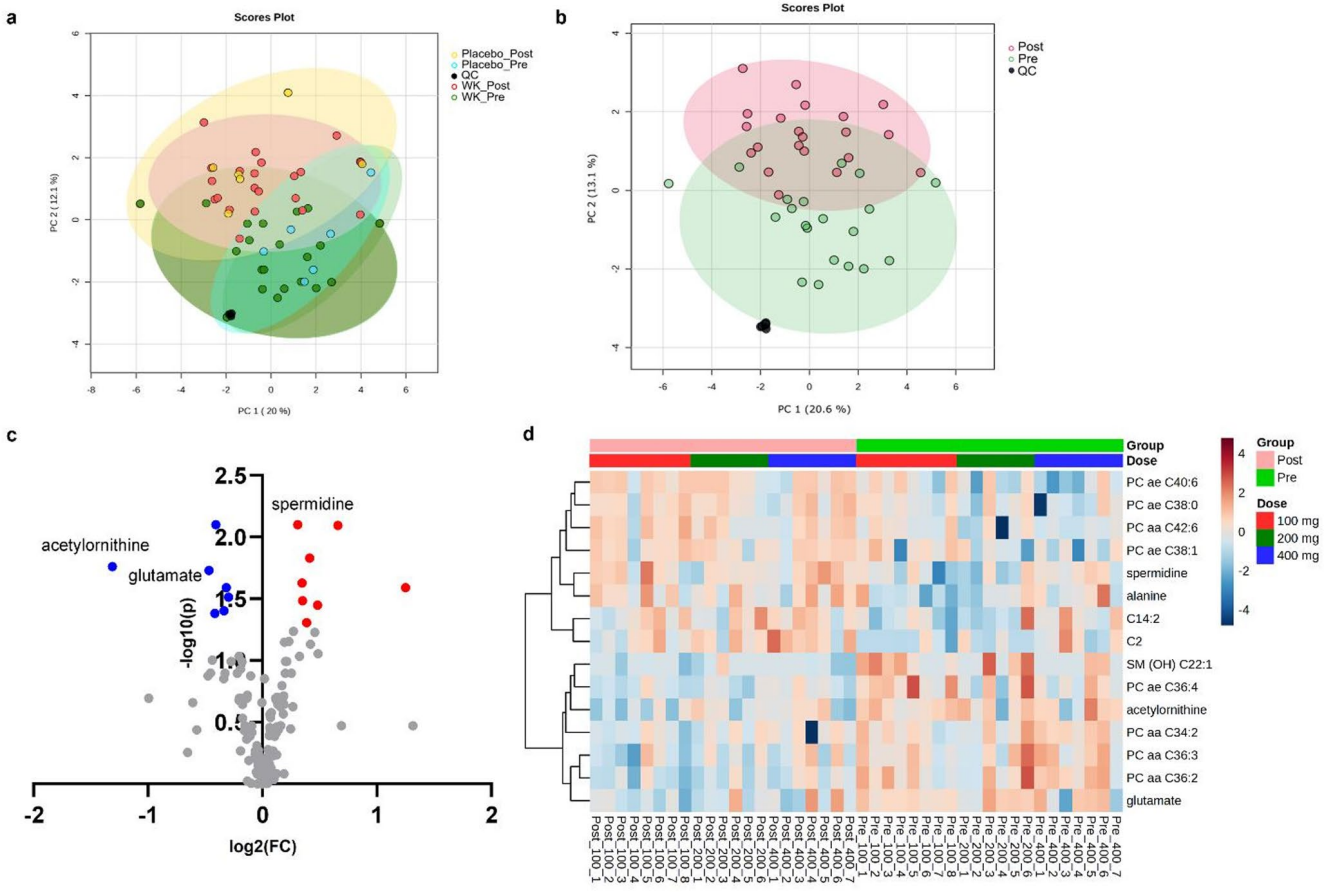
Multivariate analyses of targeted metabolomics. **(a)** PCA score plot of metabolic profiles from placebo and WK0202 treatment groups before (pre-dose) and after (post-dose) administration. **(b)** PCA score plot shows separation between pre-dose and post-dose. **(c)** Volcano plot shows the statistically significant altered metabolites in targeted analysis between pre-dose and post-dose, using a paired t-test (FDR < 0.05 and │FC│ >1.2). Red circles represent the metabolites that were upregulated, while blue circles represent the metabolites that were downregulated in post-dose. **(d)** Heat map of 15 differential metabolites in targeted analysis except placebo group between two time points

Accordingly, metabolic alterations were examined before and after drug administration. The main clusters were distinct, with changes observed between the pre- and post-dose groups in the placebo and WK0202 groups (Fig. 2a). To specifically assess the metabolic effects of WK0202, differentially abundant metabolites in the placebo group were excluded. This filtering step enabled the analysis of metabolic alterations directly attributable to WK0202 administration. Although these changes were not dose-dependent, alterations in endogenous metabolites were still observed after WK0202 administration. (Fig. S3a and Table S2). PCA showed a distinct separation between pre- and post-dose groups, regardless of the dosage, indicating significant metabolic changes following WK0202 administration (Fig. 2b).

A total of 15 metabolites showed significant changes after WK0202 administration **(**Fig. 2c and d). Eight upregulated metabolites (C2, C14:2, alanine, spermidine, PC ae C38:0, PC aa C42:6, PC ae C40:6, and PC ae C38:1) and seven downregulated metabolites (glutamate, acetylornithine, PC aa C36:2, PC aa C34:2, PC aa C36:3, PC ae C36:4, and SM (OH) C22:1) in the post-dose groups were observed (Fig. S4 and Table 1).

**Table 1:** Summary of the significant metabolites in targeted and untargeted analysis comparing pre-dose and post-dose

Metabolites	Fold Change	FDR adjusted *p* value	MSI level
*Targeted metabolomics*			
C2	1.3	0.049	-
C14:2	1.3	0.033	-
alanine	1.3	0.024	-
spermidine	1.2	0.008	-
PC ae C38:0	1.4	0.036	-
PC aa C42:6	1.6	0.008	-
PC ae C40:6	1.3	0.015	-
PC ae C38:1	2.4	0.026	-
glutamate	0.7	0.019	-
acetylornithine	0.4	0.017	-
PC aa C36:2	0.8	0.008	-
PC aa C34:2	0.8	0.04	-
PC aa C36:3	0.8	0.031	-
PC ae C36:4	0.7	0.042	-
SM (OH) C22:1	0.8	0.026	-
*Untargeted metabolomics*			
docosahexaenoic acid	2	5.53E-05	1
PC ae C40:6	1.8	0.0002	2
eicosapentaenoic acid	1.7	0.001	2
3-methyl-2-oxovaleric acid	1.6	0.004	2
3-hydroxyhexadecanoic acid	1.2	0.004	2
hydroxyphenyllactic acid	1.2	0.046	2
indole-3-propionic acid	0.2	4.15E-05	1
PC aa C36:4	0.5	0.0002	2
hippuric acid	0.4	0.0004	1
2-arachidonoylglycerol	0.7	0.0004	1
LysoPE a C18:0	0.8	0.002	2
PC aa C38:5	0.7	0.003	2
pipecolic acid	0.8	0.025	1

### Untargeted metabolomic profiling

The distribution of metabolic profiles among subjects administered varying doses of WK0202 (100, 200, and 400 mg) at both pre- and post-dose time points were analyzed (Fig. S2b). Similar to the targeted metabolomic profile, no significant metabolites were identified through the Kruskal–Wallis test. Additionally, the PERMANOVA analysis calculated a p-value of 0.470, matching the targeted metabolic profile.

**Fig. 3:**
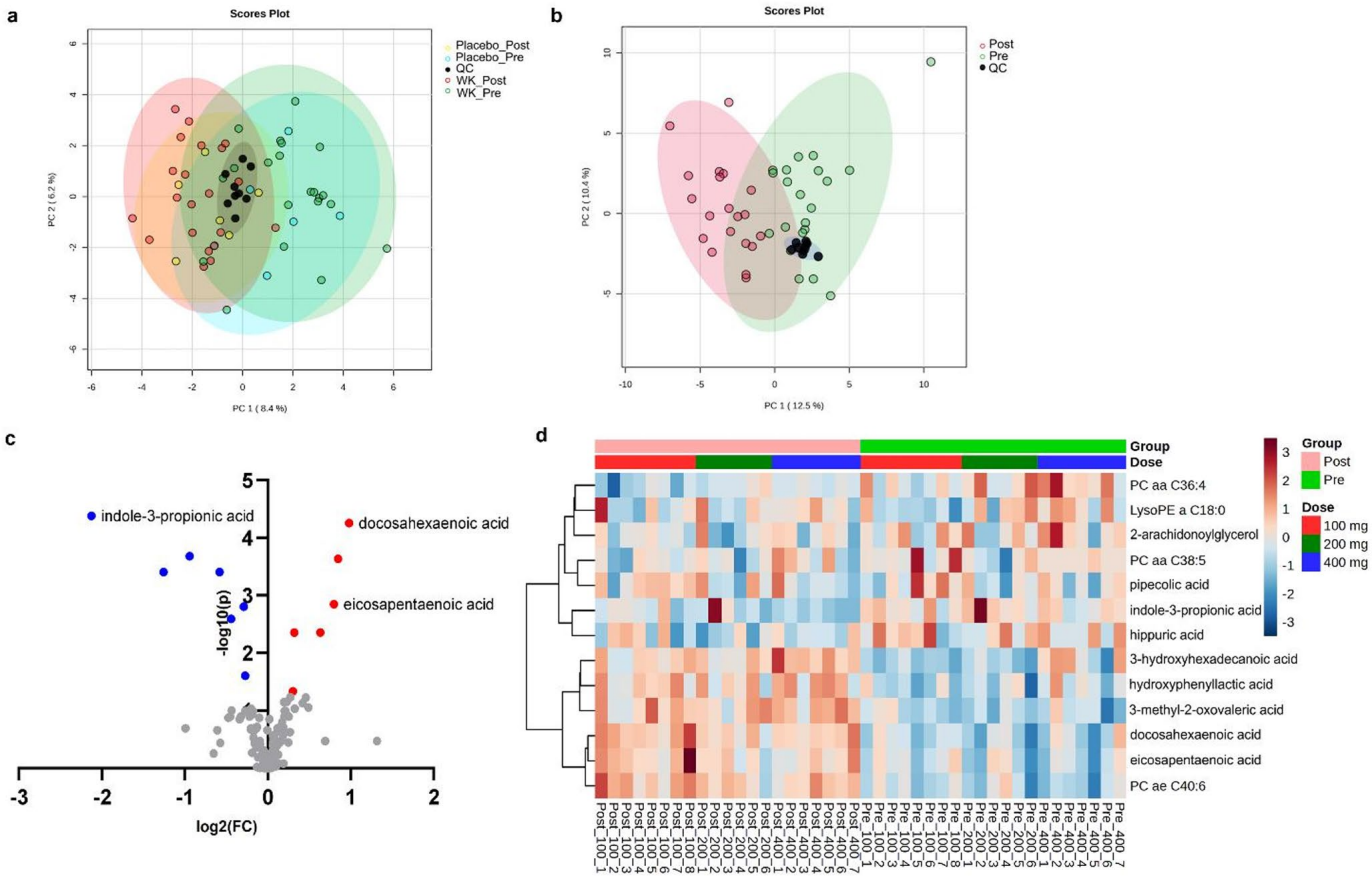
Multivariate analyses of untargeted metabolomics. **(a)** PCA score plot of metabolic profiles from placebo and WK0202 treatment groups before (pre-dose) and after (post-dose) administration. **(b)** PCA score plot shows separation between pre-dose and post-dose. **(c)** Volcano plot shows the statistically significant altered metabolites in untargeted analysis between pre-dose and post-dose, using a paired t-test (FDR < 0.05 and │FC│ >1.2). Red circles represent the metabolites that were upregulated, while blue circles represent the metabolites that were downregulated in post-dose. **(d)** Heat map of 13 differential metabolites in untargeted analysis between two time points

Among 183 metabolites were annotated by matching with databases, none of these metabolites showed significant changes in the placebo group (Fig. 3a and Fig. S3b). Separation was observed in the PCA score plot, and the gathered QCs were aligned (Fig. 3b). Among these, 13 metabolites exhibited significant alterations after WK0202 administration. These changes were identified through pairwise comparisons using a paired t-test, with an FDR p-value less than 0.05 (Fig. 3c and d). Six upregulated metabolites (docosahexaenoic acid, PC ae C40:6, eicosapentaenoic acid, 3-methyl-2-oxovaleric acid, 3-hydroxyhexadecanoic acid, and hydroxyphenyllactic acid) and seven downregulated metabolites (indole-3-propionic acid, PC aa C36:4, hippuric acid, 2-arachidonoylglycerol, LysoPE a C18:0, PC aa C38:5, and pipecolic acid) were observed in the post-dose group (Fig. S5 and Table 1). The tendency of upregulated and downregulated metabolites was consistent for each dose group in both targeted and untargeted analyses (Fig. S6 and Fig. S7).

### Network analysis and enrichment analysis

**Fig. 4:**
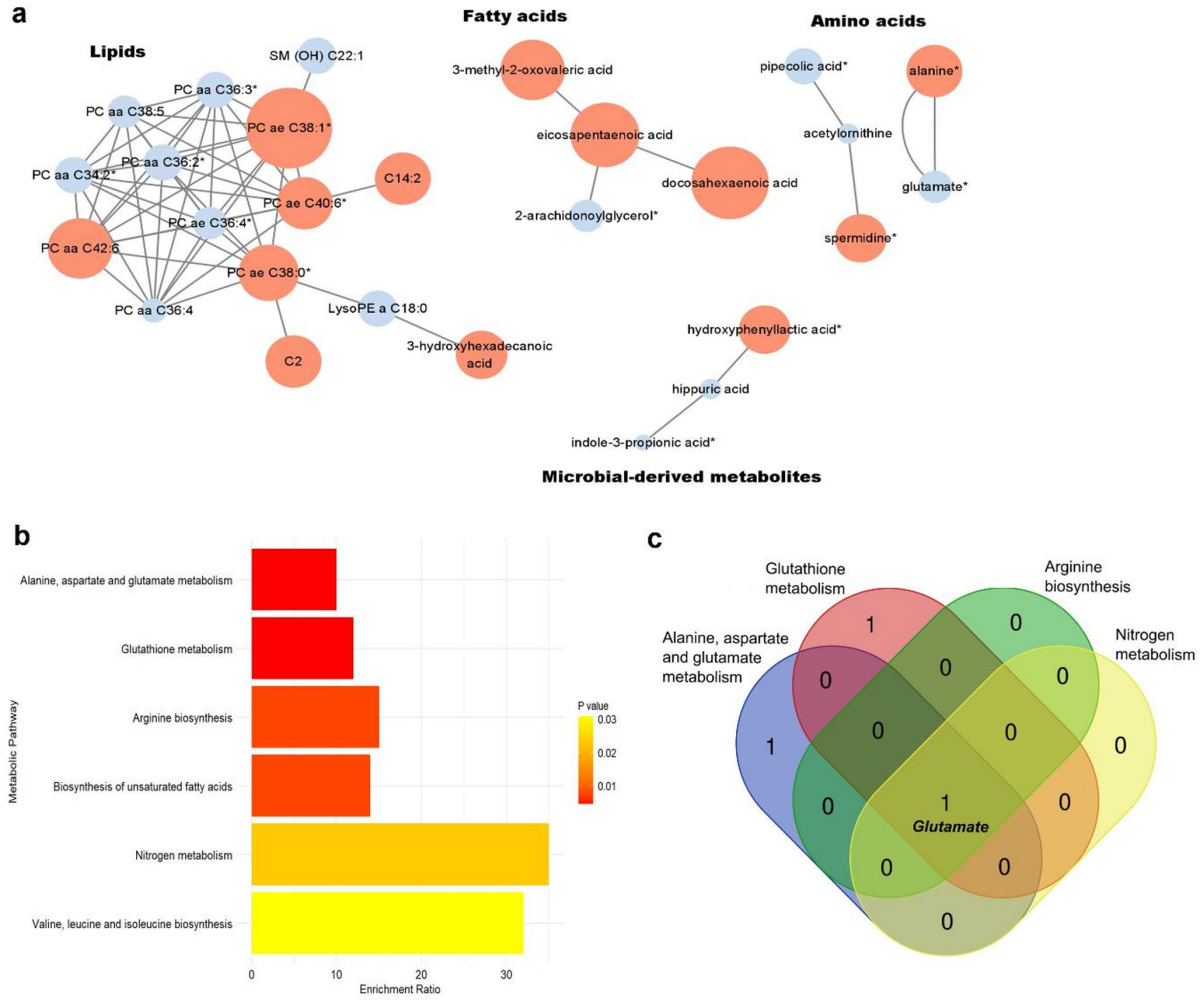
Network and pathway analysis of significantly differential metabolites. **(a)** The network analysis was performed by using Cytoscape in both targeted and untargeted analysis. The metabolites are represented as nodes and edges represent correlations between metabolites in a network. The colors of the nodes indicate up and down regulation of metabolites in post dose. Red (positive fold change) and blue (negative fold change) indicate significant metabolites that are increased or decreased after WK0202 treatments and the size of nodes represents the magnitude of fold change. * indicate metabolites that show a dose-response relationship. **(b)** Enrichment analysis of selected differential metabolites by time points in both targeted and untargeted analysis. Pathways are ranked based on enrichment ratio, with colors representing the p value. **(c)** Venn diagram of metabolite-pathway associations, highlighting the number of metabolites contributing to each metabolic pathway. This diagram includes only the metabolic pathways that involve glutamate

To better understand the relationships between altered metabolites, a network analysis was performed to group interconnected metabolites and identify their interactions. The significantly altered metabolites were categorized into four main classes: lipids, fatty acids, amino acids, and microbial-derived metabolites (Fig. 4a). The major pathways affected by WK0202 administration in the post-dose subjects were identified based on the KEGG database. The altered metabolites identified through targeted and untargeted analyses were associated with specific pathways, including alanine, aspartate, and glutamate metabolism (Fig. 4b). Among the six significantly enriched pathways, glutamate was associated with four pathways, indicating its involvement in diverse metabolic processes (Fig. 4c). Notably, glutamate was a key node across several pathways including alanine, aspartate and glutamate metabolism, nitrogen metabolism, arginine biosynthesis, and glutathione metabolism, highlighting its role as a central metabolite in metabolic regulation. The observed changes in glutamate levels suggested that WK0202 administration may influence these interconnected pathways, further supporting its systemic metabolic effects.

### Glutamate and its role in WK0202

Given that NQO1 is important for maintaining redox homeostasis via NAD+/NADH balance (Kim et al., 2013), genetic variations in NQO1 might affect metabolic pathways reliant on this balance. Therefore, to investigate the impact of NQO1 genotype on metabolism, Spearman correlation analysis was performed on post/pre-dose concentration ratios of significantly altered metabolites and NQO1 genotype. Most metabolites did not show differ significantly by NQO1 genotype (Fig. S8 and Fig. S9). However, glutamate exhibited a statistically significant correlation (*r* = 0.466, *p* = 0.03).

**Fig. 5:**
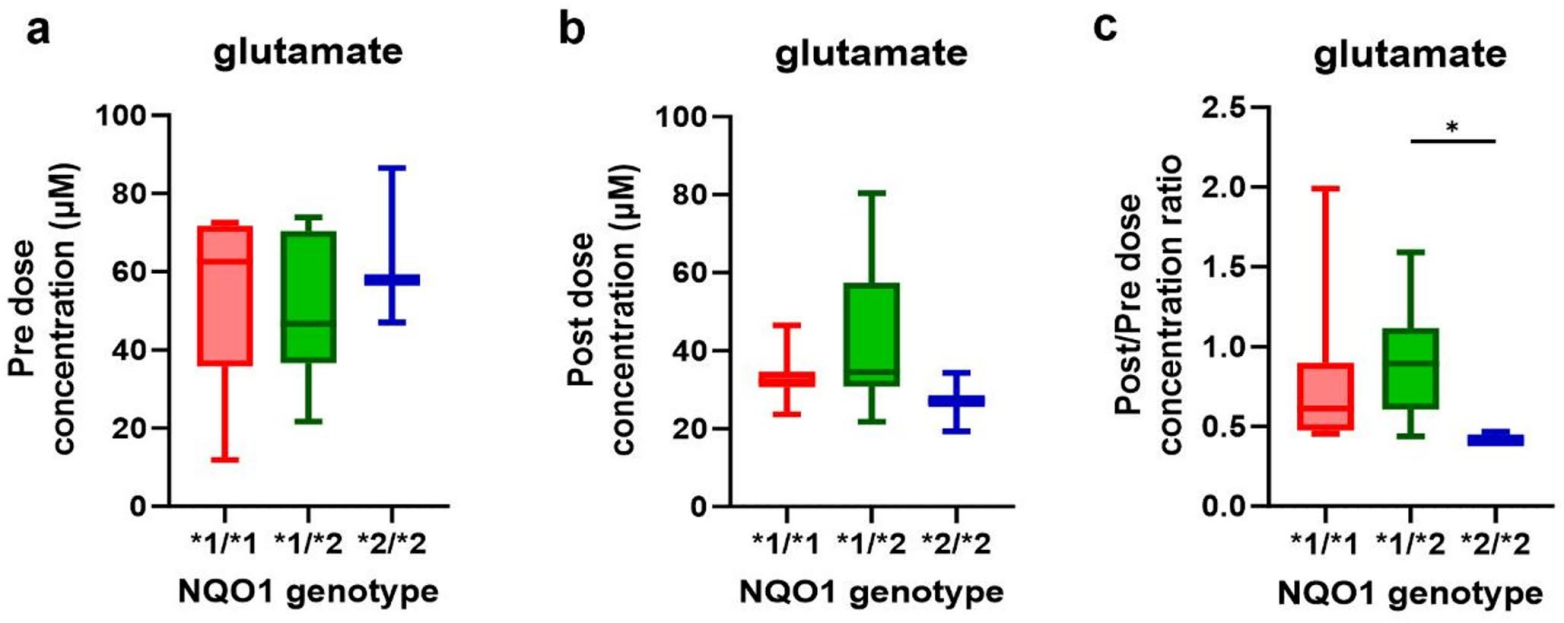
Box plots of glutamate levels by NQO1 genotype. **(a-c)** Box plots show the pre-dose **(a)**, post-dose **(b)**, and post/pre dose concentration ratio **(c)** of glutamate levels by NQO1 genotypes (*1/*1, *n* = 7; *1/*2, *n* = 11; *2/*2, *n* = 3)

Since glutamate is central in energy metabolism and oxidative stress regulation, the effect of NQO1 genotype on glutamate levels after WK0202 administration was investigated further. Though pre-dose levels showed increase levels of glutamate but post-dose levels showed decrease levels of glutamate in every NQO1 genotypes (Fig. 5a and b). The post/pre-dose concentration ratio varied significantly among NQO1 genotypes **(**Fig. 5c**)**, with the *2/*2 genotype, which is associated with reduced NQO1 enzymatic activity, showing the greatest decrease. While most metabolites did not show significant differences across NQO1 genotypes, glutamate levels were uniquely affected. This suggested that WK0202 induced metabolic responses are genotype-independent, except for glutamate.

## Discussion

**Fig. 6:**
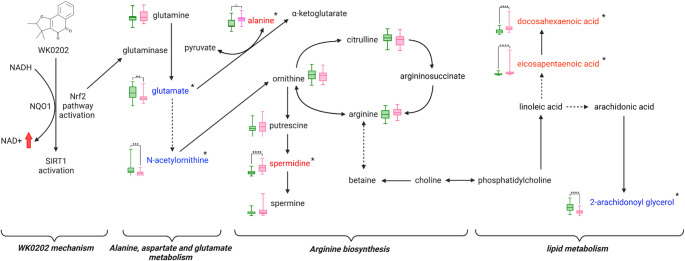
Major metabolic pathways influenced by WK0202 administration. Proposed metabolic pathways affected by WK0202 administration. Red letters with * and blue letters with * indicate significant metabolites that are increased or decreased after β-lapachone derivative treatment in healthy volunteers. (pink box plots: post-dose concentration of metabolite, green box plots: pre-dose concentration of metabolite)

This first-in-human study provides an initial assessment of the pharmacodynamic effects and mechanism of WK0202 by comparing pre- and post-dose metabolomic profiles using both targeted and untargeted approaches. The findings of this study demonstrate that WK0202 administration induces notable changes in alanine, aspartate, and glutamate metabolism, arginine biosynthesis, and lipid metabolism **(**Fig. 6**)**. Collectively, this study improves the understanding of WK0202’s potential mechanisms and pharmacodynamic biomarker candidates.

Significant changes were observed in alanine, aspartate and glutamate metabolism following WK0202 administration, including an increase in glutamine levels and a corresponding decrease in glutamate levels. Although the change in glutamine was not statistically significant, this pattern aligns with previous research that reported similar metabolic alterations upon β-lapachone treatment (Silvers et al., 2017). Despite the differences in their primary mechanisms of action, the observed metabolic shifts suggest both compounds may influence glutamate metabolism and glutaminase activity. Although the current study did not directly measure glutaminase activity, prior research suggests that activation of the Nrf2 pathway may influence glutaminase activity (Hamada et al., 2021; Sayin et al., 2017). This may contribute to the observed metabolic changes. Given that WK0202 primarily activates NQO1, the observed metabolic changes may be related to Nrf2-related pathways involved in antioxidant and anti-inflammatory responses (Andreasen et al., 2009; Hnia et al., 2008; Kim et al., 2015; Nemati et al., 2019).

After WK0202 administration, glutamate levels were decrease across all NQO1 genotypes, with a particularly limited regulation observed in the *2/*2 genotype. The reduction of glutamate across all genotypes and dose groups may reflect decreased glutaminase activity, which aligns with previous studies suggesting that glutaminase inhibition could serve as a therapeutic strategy (Song et al., 2017). Recent studies showed that compounds with dual glutaminase inhibition and Nrf2 activation activities effectively prevent diseases such as chemotherapy-induced peripheral neuropathy (Foster et al., 2025). A reduction of glutamate may contribute to decreased pain and activation of the Nrf2 signaling pathway is known to alleviate oxidative stress (Foster et al., 2025). These observations suggest that glutamate-related metabolic pathways may serve as early indicators of WK0202’s pharmacological response and mechanism of action, requiring investigation and validation.

One of the most significant findings of our analysis was the alteration in arginine biosynthesis following WK0202 administration. Arginine supplementation initiates the Nrf2 pathway, leading to an increase in antioxidant expressions driven by the antioxidant response element through the Nrf2 pathway (Liang et al., 2018; Ma et al., 2022; Perez de la Lastra et al., 2023). Spermidine-induced Nrf2 activation enhances antioxidant gene expression and anti-inflammatory responses, supporting increased polyamine metabolism and activation of the Nrf2 pathway (Aihara et al., 2023; Dasdelen et al., 2023; Jiang et al., 2021; Liu et al., 2019). Our data indicate an overall increase in the metabolism of polyamines and arginine, suggesting that WK0202 modulates cellular redox homeostasis by activating the Nrf2 pathway. WK0202 affects key metabolic pathways involved in the biosynthesis of arginine and polyamines by activating NQO1 and inducing the Nrf2 pathway. Collectively, the activation of these pathways potentially contributes to the antioxidant and anti-inflammatory responses.

We expanded on existing research showing the role of Nrf2 in regulating key genes involved in lipid metabolism, suggesting that WK0202 may interact with lipid pathways (Huang et al., 2010). Notably, upregulation of lipids may indicate enhanced lipid remodeling, which is critical for cellular protection and repair mechanisms (Neis et al., 2017; Santos & Preta, 2018; Yu et al., 2021). Previous studies have indicated that phosphatidylcholines (PCs) contribute to cellular homeostasis by modulating lipid metabolism under stress conditions (Tan et al., 2020). These metabolic alterations are consistent with the known mechanisms of WK0202, which activate the NQO1 and Nrf2 pathways, resulting in specific metabolic responses. Our findings suggest the possible involvement of several lipids in the Nrf2 pathway, notably docosahexaenoic acid and eicosapentaenoic acid. These omega-3 fatty acids upregulate the Nrf2-mediated antioxidant response and may contribute to redox homeostasis (Lee et al., 2023), highlighting a possible link between lipid metabolism and oxidative stress regulation. Upregulation of docosahexaenoic acid and eicosapentaenoic acid, both known for their anti-inflammatory and antioxidant properties, suggests that WK0202 may modulate lipid-associated metabolic pathways (Kim & Chung, 2007; Lee et al., 2023; Tatsumi et al., 2019).

However, this study has some limitations that need to be acknowledged. Our analysis was limited to healthy subjects and a small sample size, which prevented us from directly analyzing the correlation between the actual therapeutic effects of WK0202 and changes in endogenous metabolites in patients. Additionally, only healthy male participants were included, limiting the applicability of our findings to female populations. Given that baseline metabolite levels may vary among humans, caution is required when interpreting our findings (Suhre et al., 2011). Furthermore, while our metabolomic analysis revealed significant alterations in key metabolic pathways, the expression of inflammatory biomarkers, NQO1, and other Nrf2-related enzymes were not directly measured. To validate and extend these observations, further studies with larger cohorts, a broader range of participants including both sexes and patient populations, and a robust sampling will be essential. To strengthen the mechanistic interpretation, future studies should include direct measurements of NQO1 and Nrf2-related enzymes. These future studies will help confirm whether the observed metabolic changes truly reflect the pharmacodynamic effects of WK0202 and whether these candidate metabolites can serve as reliable biomarkers in clinical practice.

Overall, this study explored metabolic changes induced by WK0202 administration, focusing on the relationship between NQO1 activation and the Nrf2 pathway. Mechanistically, WK0202 may activate NQO1, increasing NAD + levels and activating SIRT1. Metabolomics profiling identified significant changes in alanine, aspartate, and glutamate metabolism, arginine biosynthesis, and lipid metabolism. These findings suggest a possible involvement of the Nrf2 pathway in modulating glutaminase activity and related metabolic responses. The results of this study offer preliminary insights into the potential mechanism of action and pharmacodynamic biomarker candidates of WK0202. To validate these findings, larger scale clinical studies with broader populations will be necessary.

## Supplementary Information

Below is the link to the electronic supplementary material.

Supplementary Material 1

## Data Availability

Raw data are available at the Korea BioData Station (K-BDS, https://kbds.re.kr/) under accession ID KAP240812.

## Abbreviations

ALT: Alanine aminotransferase
AMPK: 5’ adenosine monophosphate-activated protein kinase
AST: Aspartate aminotransferase
BMI: Body mass index
CPK: Creatine phosphokinase
eGFR: Estimated glomerular filtration rate
FDR: False discovery rate
LOD: Limit of detection
NAD+: Nicotinamide adenine dinucleotide
NF-κB: Nuclear factor kappa B
NQO1: NAD(P)H quinone dehydrogenase 1
Nrf2: Nuclear factor erythroid-derived 2-related factor 2
PCA: Principal component analysis
PC: Phosphatidylcholine
QC: Quality control
UPLC: Ultra-high-performance liquid chromatography
