# Supplementary material for: Pharmacometabolomics uncovers key metabolic changes in the first-in-human study of β-lapachone derivative
Source: Metabolomics. 2025 Aug 19;21(5):122. doi: 10.1007/s11306-025-02332-1 (PMC12364990; doi:10.1007/s11306-025-02332-1)
Supplement: Supplementary file 1 — Supplementary Material 1 [file 11306_2025_2332_MOESM1_ESM.docx]

**Supplementary materials**

**Pharmacometabolomics uncovers key metabolic changes in the first-in-human study of β-lapachone derivative**

Yeonseo Jang^a†^, Jihyun Kang^a,c,^**^^[[1]](#footnote-1)^^**^†^, Yufei Li^a^, Woori Chae^a,c^, Eunsol Yang^a,2^, SeungHwan Lee^a^ and Joo-Youn Cho^a,b,c,d*^

^a^Department of Clinical Pharmacology and Therapeutics, Seoul National University College of Medicine and Hospital, Seoul 03080, Korea

^b^Department of Biomedical Sciences, Seoul National University College of Medicine and Hospital, Seoul 03080, Korea

^c^Kidney Research Institute, Seoul National University Medical Research Center, Seoul 03080, Korea.

^d^Seoul National University, Seoul 08826, Republic of Korea.

† These authors equally contributed to this work.

*Corresponding author: [joocho@snu.ac.kr](mailto:joocho@snu.ac.kr)


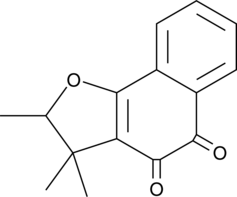


**Figure S1. Chemical structure of WK0202.**

**
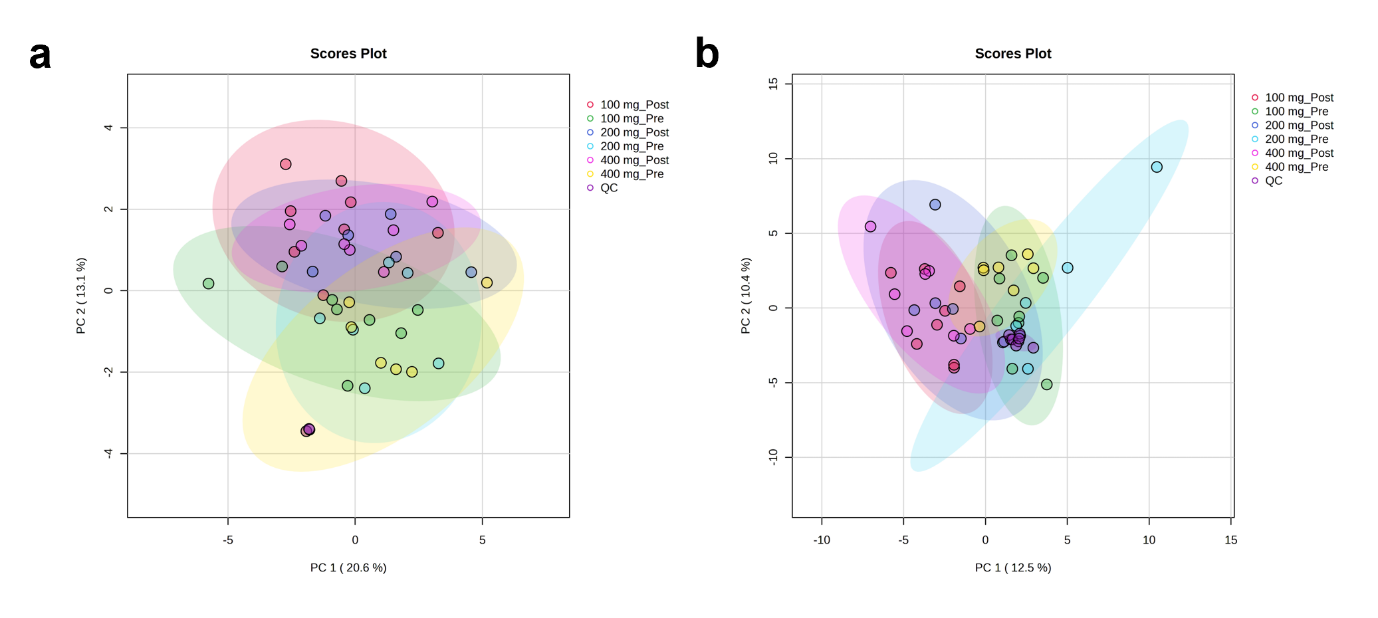
**

**Figure S2. PCA score plots show no separation between doses.**

Each point represents an individual sample, with colors by the groups: red (100 mg_Post), green (100 mg_Pre), blue (200 mg_Post), cyan (200 mg_Pre), pink (400 mg_Post), yellow (400 mg_Pre), and purple (QC).


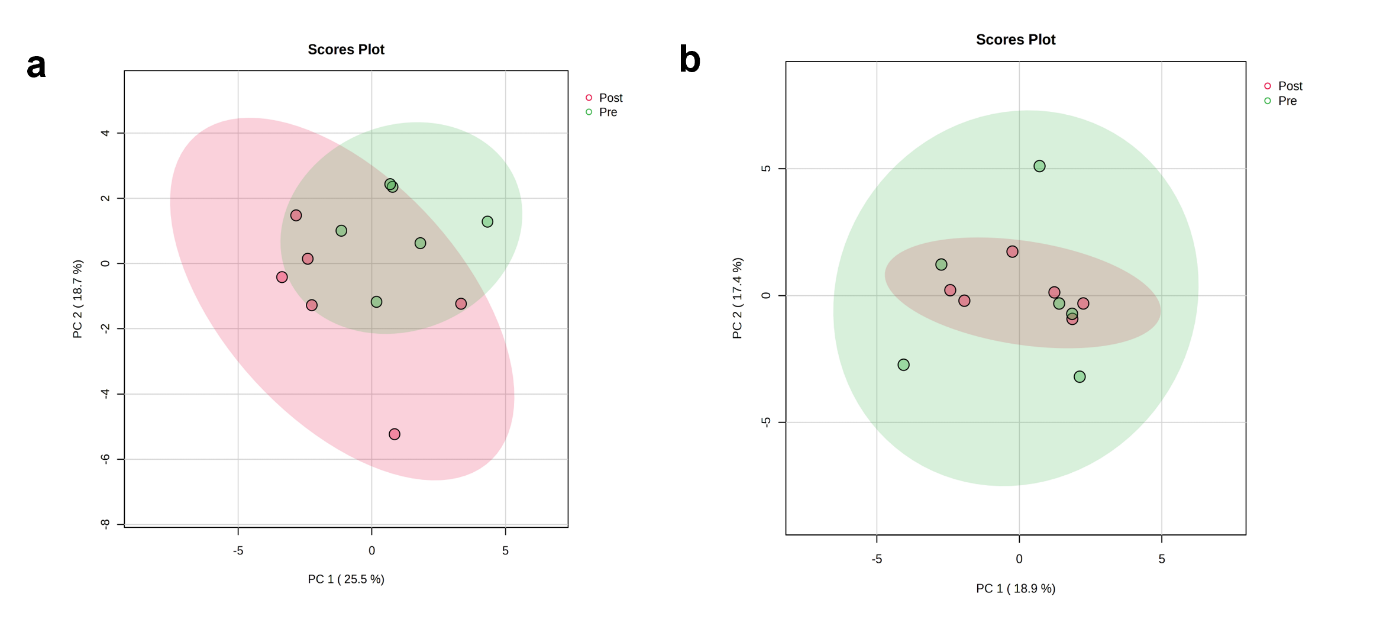


**Figure S3. PCA score plots of placebo group in targeted analysis (a) and untargeted analysis (b)**

**
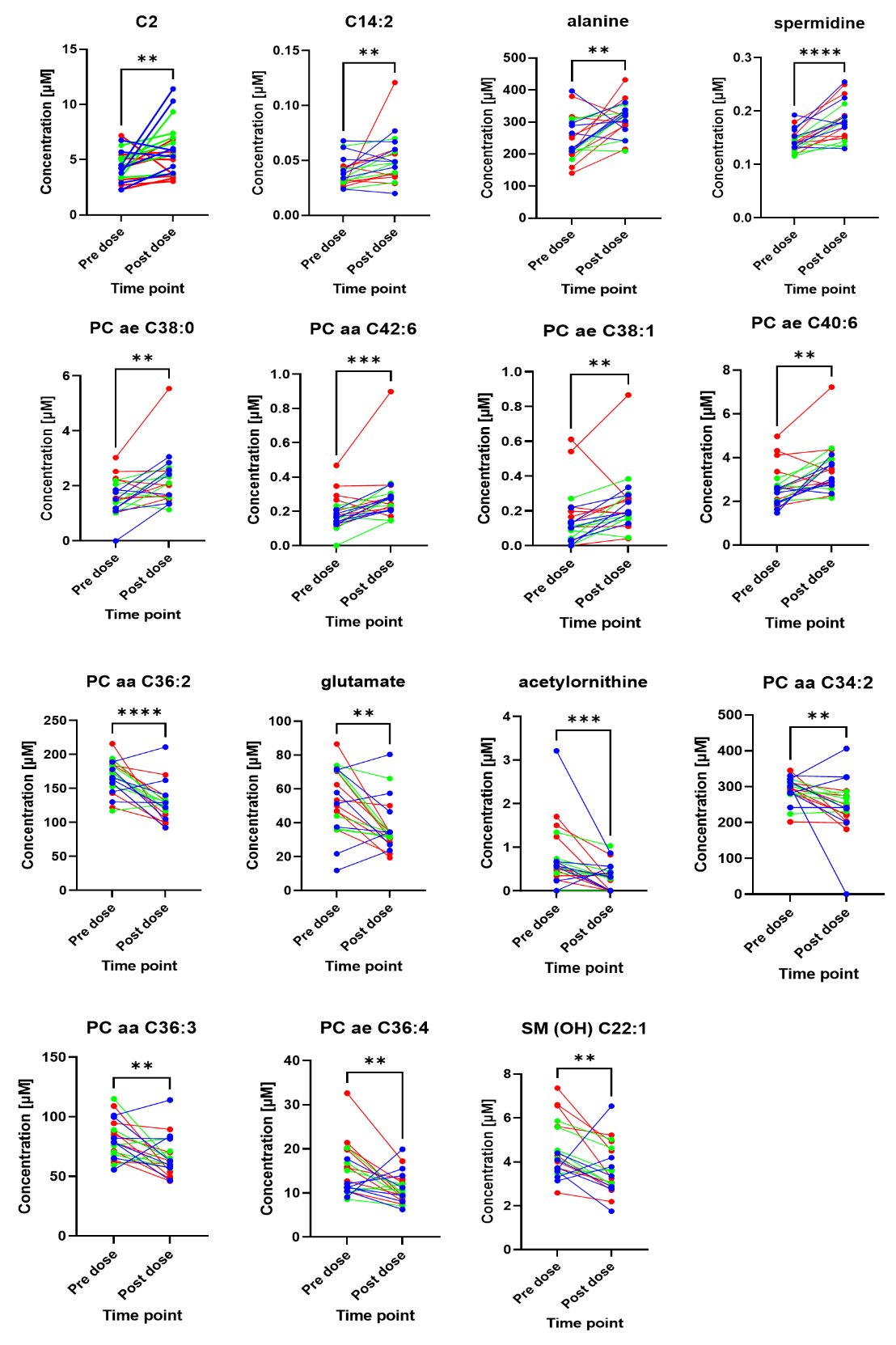
**

**Figure S4. Spaghetti plots of the 15 metabolites comparing pre-dose and post-dose**

C2, C14:2, alanine, spermidine, PC ae C38:0, PC aa C42:6, PC ae C38:1, and PC ae C40:6 were upregulated and PC aa C36:2, glutamate, acetylornithine, PC aa C34:2, PC aa C36:3, PC ae C36:4, and SM (OH) C22:1 were downregulated in post-dose. FDR adjusted p value was calculated using the paired t-test whose significance is expressed as p<0.05.

*p < 0.05, **p < 0.01, *** p < 0.005, **** p < 0.0001. red : 100 mg, green : 200 mg, blue : 400 mg.


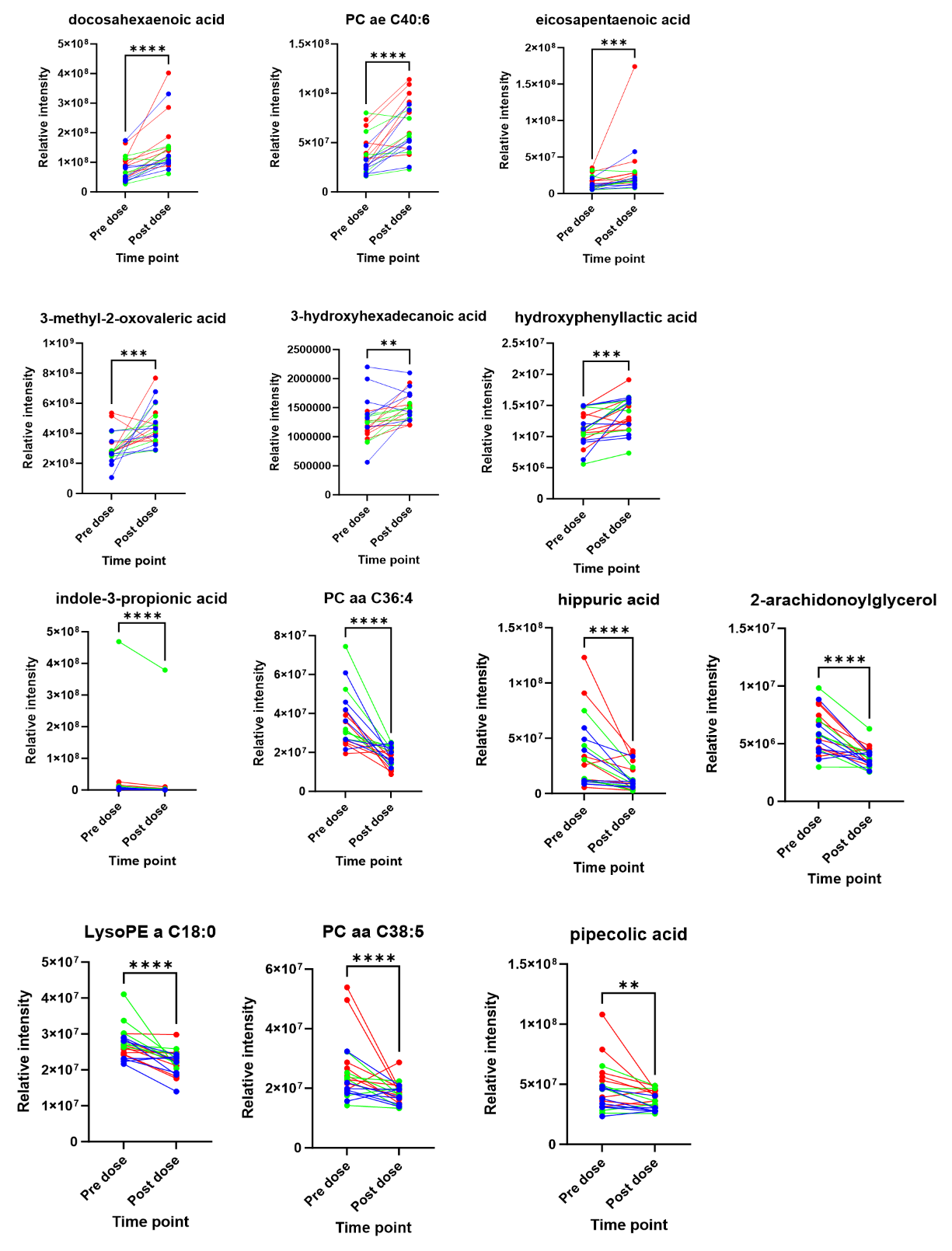


**Figure S5. Spaghetti plots of the 13 metabolites comparing pre-dose and post-dose in untargeted analysis.**

Docosahexaenoic acid, PC ae C40:6, eicosapentaenoic acid, 3-methyl-2-oxovaleric acid, 3-hydroxyhexadecanoic acid, and hydroxyphenyllactic acid were found to be upregulated and indole-3-propionic acid, PC aa C36:4, hippuric acid, 2-arachidonoylglycerol, LysoPE a C18:0, PC aa C38:5, and pipecolic acid were downregulated in post-dose (15 d 0 h). FDR adjusted p value was calculated using the paired t-test whose significance is expressed as p<0.05.

*p < 0.05, **p < 0.01, *** p < 0.005, **** p < 0.0001. red : 100 mg, green : 200 mg, blue : 400 mg.

**alanine**


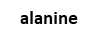


**Figure S6. Box plots of the top 15 metabolites in targeted analysis.**

FDR adjusted p value was calculated using the paired t-test at the same dose whose significance is expressed as p<0.05. (*p < 0.05, **p < 0.01)

**
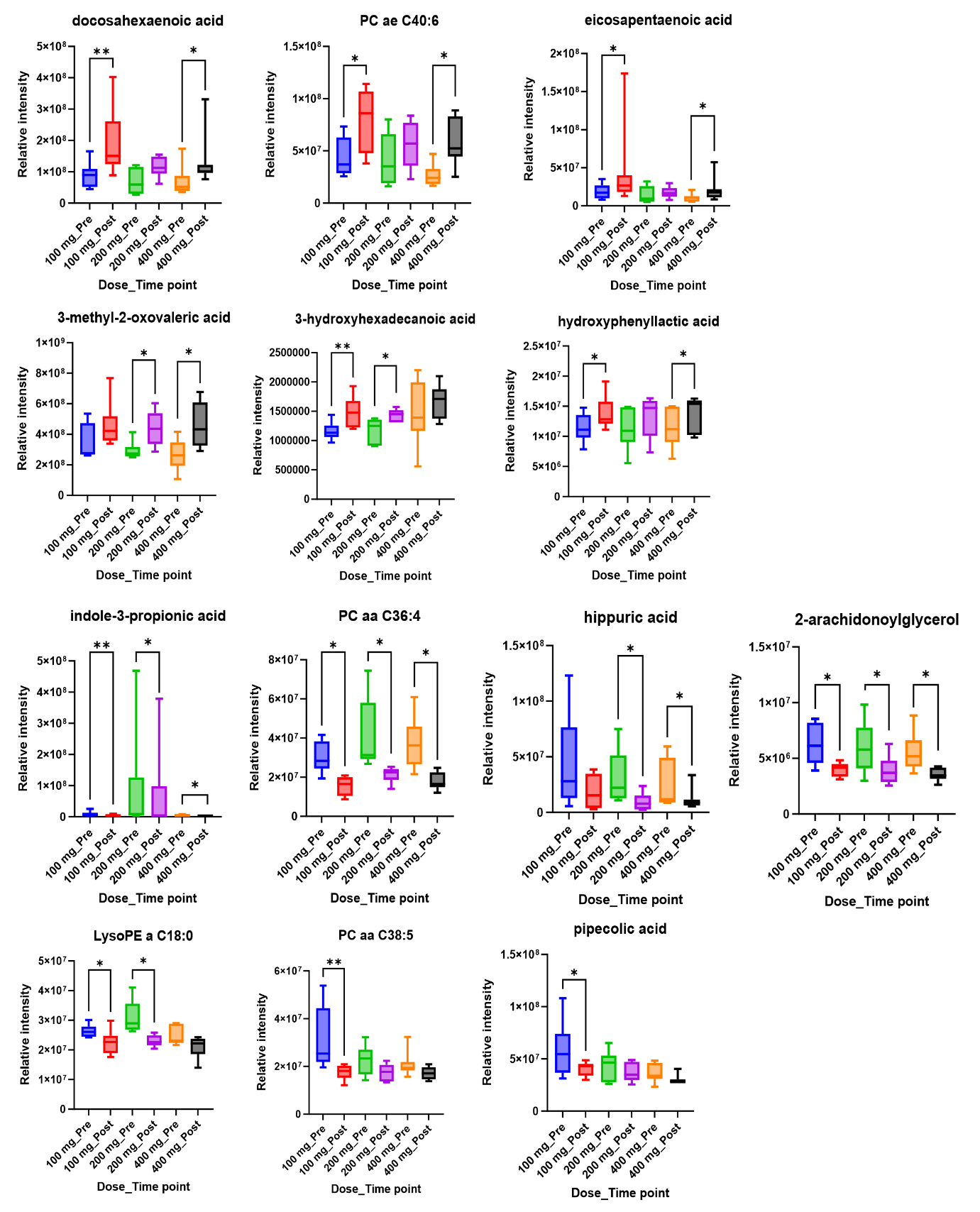
**

**Figure S7. Box plots of 13 metabolites in untargeted analysis.**

FDR adjusted p value was calculated using the paired t-test at the same dose whose significance is expressed as p<0.05. (*p < 0.05, **p < 0.01)


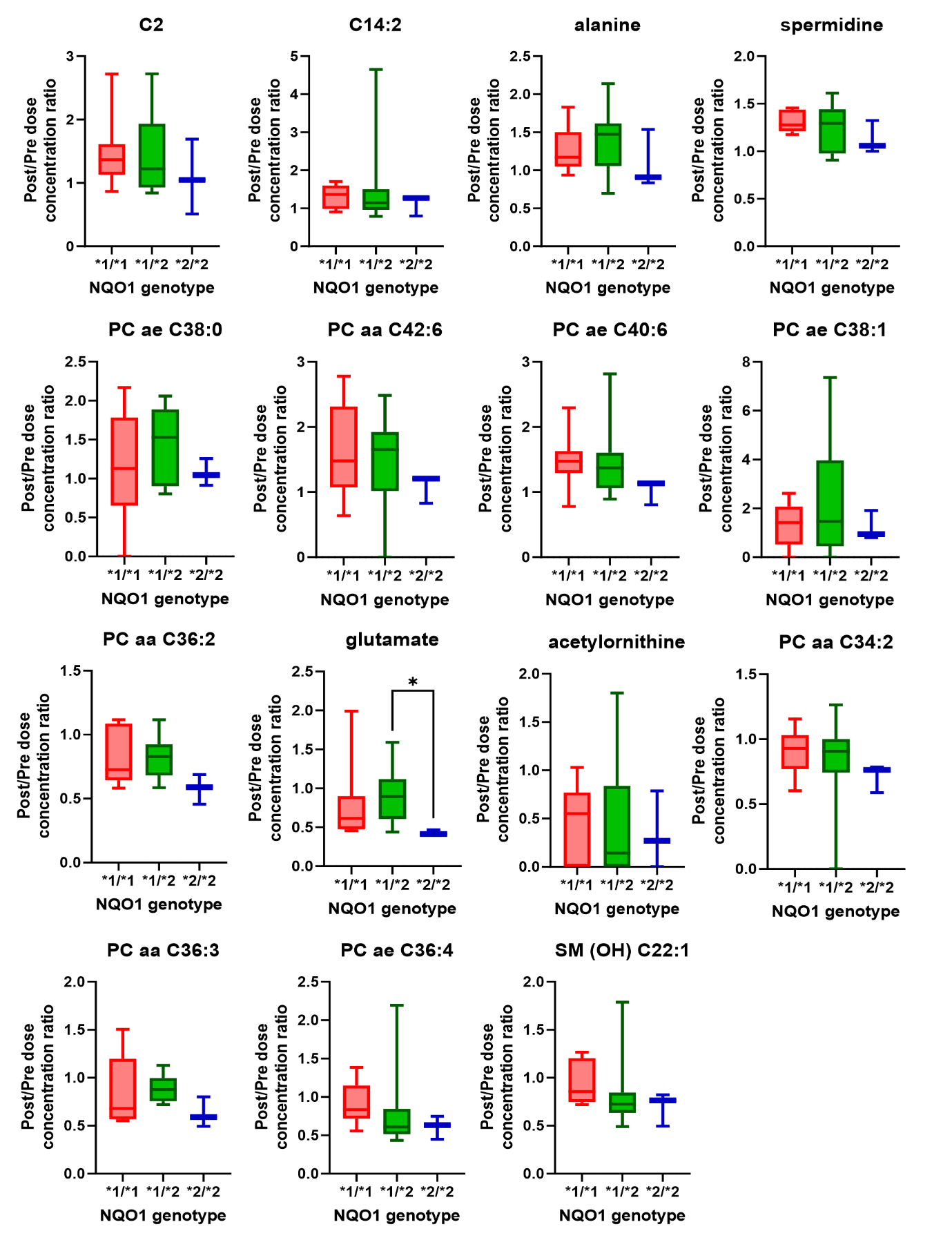


**Figure S8. Box plots of 15 metabolites in targeted analysis.**

The y-axis represents the ratio of post-dose to pre-dose concentrations for each metabolite. The Kruskal-Wallis test was used to assess NQO1 genotype and metabolites relationship, and FDR-adjusted p-value was calculated for each metabolite at NQO1 genotype groups. (*p < 0.05)

**
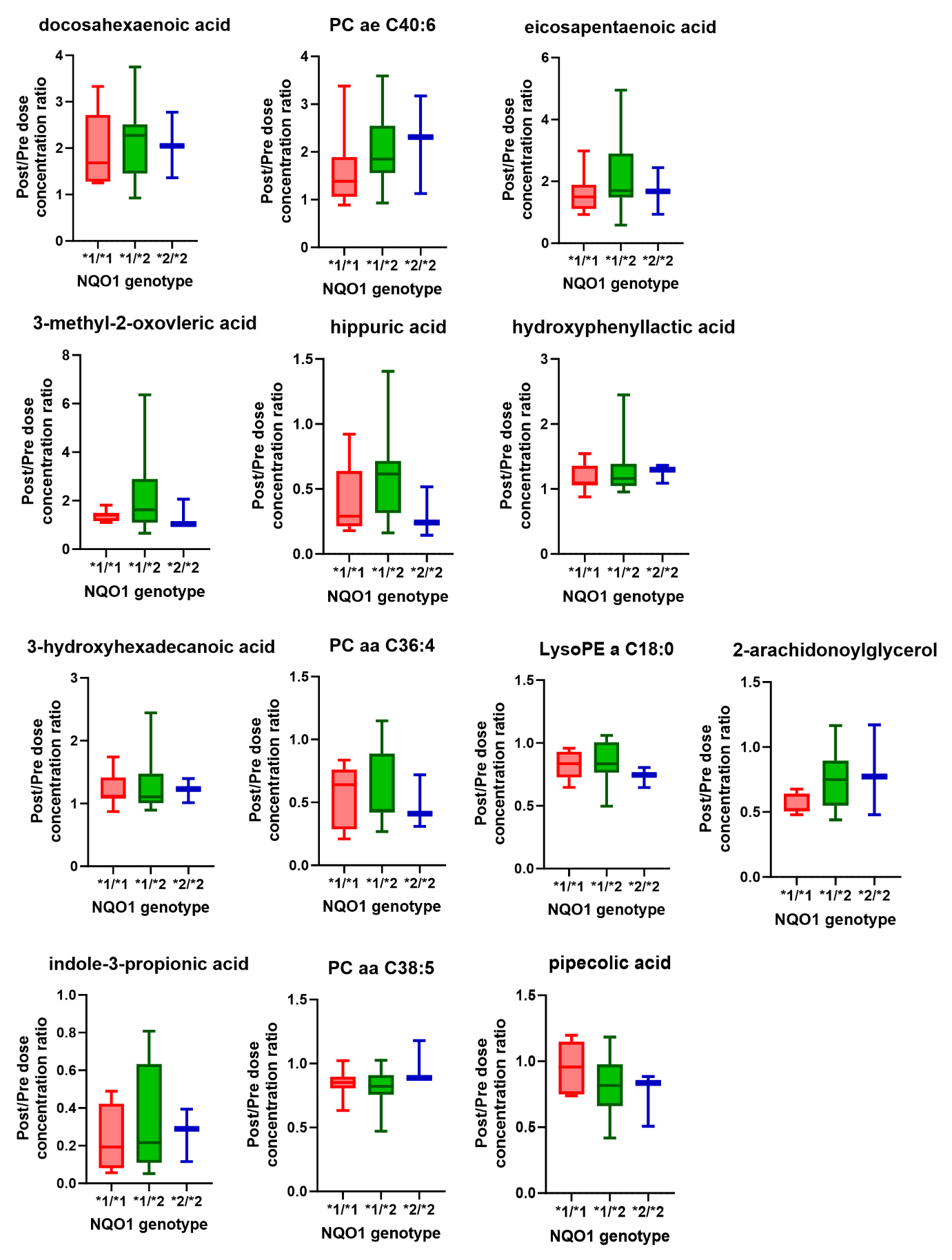
**

**Figure S9. Box plots of 13 metabolites in untargeted analysis.**

The y-axis represents the ratio of post-dose to pre-dose concentrations for each metabolite. The Kruskal-Wallis test was used to assess NQO1 genotype and metabolites relationship, and FDR-adjusted p-value was calculated for each metabolite at NQO1 genotype groups.

**Table S1. Demographic characteristics of the study participants.**

|  | WK0202  100 mg  (N=8) | WK0202  200 mg  (N=6) | WK0202  400 mg  (N=7) | Placebo  (N=6) | P  P value |
| --- | --- | --- | --- | --- | --- |
| Age (years) | 30.50 (4.96) | 34.38 (6.82) | 33.44 (7.78) | 30.57 (6.63) | > 0.05 |
| Height (cm) | 172.63 (3.25) | 172.38 (4.44) | 171.38 (3.49) | 173.57 (8.50) | > 0.05 |
| BMI (kg/$\boldsymbol{m}^{\boldsymbol{2}}$) | 24.19 (2.61) | 23.60 (1.85) | 23.76 (2.14) | 23.45 (2.61) | > 0.05 |
| NQO1 genotype, n (%) |  |  |  |  |  |
| *1/*1 | 2 (25) | 3 (50) | 2 (28.57) | - |  |
| *1/*2 | 4 (50) | 3 (50) | 4 (57.14) | - |  |
| *2/*2 | 2 (25) | 0 (0) | 1 (14.29) | - |  |

The age, height, BMI data in the demographic table are reported as mean (SD).

SD : standard deviation, BMI : body mass index

**Table S2. Summary of the 22 metabolites changed in placebo group based on p value<0.05 by paired t-test.**

| Metabolites | Fold Change | p value | Up/Down |
| --- | --- | --- | --- |
| LysoPC a C18:2 | 1.8 | 0.001 | ↑ |
| PC ae C36:0 | 0.5 | 0.001 | ↓ |
| PC aa C42:1 | 0.7 | 0.003 | ↓ |
| PC ae C34:0 | 0.6 | 0.004 | ↓ |
| PC aa C42:0 | 0.7 | 0.005 | ↓ |
| PC aa C36:0 | 0.6 | 0.007 | ↓ |
| PC aa C38:6 | 0.6 | 0.010 | ↓ |
| ADMA | 1.2 | 0.013 | ↑ |
| ornithine | 1.3 | 0.013 | ↑ |
| PC ae C34:2 | 1.4 | 0.013 | ↑ |
| PC aa C42:2 | 0.7 | 0.019 | ↓ |
| PC aa C30:0 | 0.9 | 0.023 | ↓ |
| isoleucine | 0.8 | 0.025 | ↓ |
| PC aa C38:1 | 0.5 | 0.026 | ↓ |
| PC aa C38:0 | 0.7 | 0.029 | ↓ |
| PC aa C40:2 | 0.7 | 0.032 | ↓ |
| SM C26:1 | 0.7 | 0.036 | ↓ |
| PC aa C40:6 | 0.5 | 0.041 | ↓ |
| PC ae C40:5 | 0.8 | 0.042 | ↓ |
| citrulline | 1.3 | 0.045 | ↑ |
| PC ae C40:2 | 0.8 | 0.045 | ↓ |
| lysoPC a C18:1 | 1.3 | 0.047 | ↑ |
| PC aa C40:6 | 0.7 | 0.047 | ↓ |

↑ indicates up-regulation and ↓ indicates down-regulation in post-dose.

**Table S3. Summary of non-significant metabolites in major pathway based on p value<0.05 by paired t-test.**

| Metabolites | Fold Change | p value | Up/Down |
| --- | --- | --- | --- |
| ornithine | 0.9 | 0.4 | ↓ |
| citrulline | 0.9 | 0.04 | ↓ |
| glutamine | 1.05 | 0.2 | ↑ |
| arginine | 1.1 | 0.1 | ↑ |
| putrescine | 1.2 | 0.2 | ↑ |
| spermine | 1.04 | 0.1 | ↑ |

↑ indicates up-regulation and ↓ indicates down-regulation in post-dose.

1. Department of Chemistry, Washington University in St.Louis, St.Louis, MO, United States of America.

   2 Department of Bioengineering and Therapeutic Sciences, University of California, San Fransisco, San Fransisco, CA, United States of America. [↑](#footnote-ref-1)
